# Violence against Emergency Nurses in Kermanshah-Iran: Prevalence and Associated Factors

**DOI:** 10.1155/2023/9362977

**Published:** 2023-01-13

**Authors:** Maryam Janatolmakan, Alireza Abdi, Shahab Rezaeian, Negin Framarzi Nasab, Alireza Khatony

**Affiliations:** ^1^Social Development and Health Promotion Research Center, Health Institute, Kermanshah University of Medical Sciences, Kermanshah, Iran; ^2^School of Nursing and Midwifery, Kermanshah University of Medical Sciences, Kermanshah, Iran; ^3^Infectious Diseases Research Center, Kermanshah University of Medical Sciences, Kermanshah, Iran

## Abstract

**Background:**

Violence against emergency nurses is a global concern with undesirable physical and psychological consequences. This study was conducted to investigate the characteristics of physical and verbal violence against emergency nurses in Iran.

**Methods:**

In this cross-sectional study, 150 nurses working in seven hospitals affiliated to Kermanshah University of Medical Sciences were included in the study using the stratified random sampling method. The data collection tools included a personal information form and a researcher-made questionnaire. Violence-related characteristics were assessed using descriptive statistics. Logistic regression was used to identify factors related to physical and verbal violence.

**Results:**

The frequency rates of physical and verbal violence during the past 12 months were equal to 62% (*n* = 93) and 94.7% (*n* = 142), respectively. In both types of physical violence (49.5%, *n* = 46) and verbal violence (40.4%, *n* = 57), the nursing station was the most common place of violence. In both physical (*n* = 40, 43.0%) and verbal violence (*n* = 101, 71.1%), the most common perpetrator was the patient's family. Most physical violence (57.0%, *n* = 53) and verbal violence (35.2%, *n* = 50) occurred in the night shifts. No statistically significant relationship was found between physical and verbal violence and gender, age, marital status, type of employment, and work experience. *Discussion*. The results indicate the seriousness of workplace violence against nurses. It is necessary to adopt a global approach along with providing sufficient manpower and psychological empowerment of nurses. Further studies with a forward-looking approach are suggested.

## 1. Introduction

Workplace violence (WPV) against emergency nurses is a global problem [1]. According to the International Labor Organization, WPV includes ill-treatment and threatening or abusive behavior, including physical or psychological violence [2–6]. WPV varies from verbal aggression and sexual harassment to physical assault, of which the verbal type is the most common form of healthcare-related aggression, and about two-thirds of nurses worldwide have experienced this type of violence [7].

By definition, physical violence is the intentional use of physical force against an individual or a group that results in physical or psychological harm. Verbal violence is a behavior that leads to humiliation or disrespect for a person's dignity [8]. Evidence suggests that healthcare workers, especially nurses, are the most vulnerable to violence due to their daily exposure to patients and their families [9]. In this regard, the results of a study in Pakistan (2021) showed 29 cases of violence against doctors and nurses committed by the patients' relatives from April to August 2020. The reasons for the violence were the deaths of patients, the nonadmission of patients, and the lack of trust in medical personnel [10].

In a study in Kenya (2021), the prevalence of violence against emergency nurses in one year was 73.2%, and the perpetrators of most of the violence were patients and their relatives. The most common type of violence was verbal abuse [11]. In a study conducted in Saudi Arabia (2022), the prevalence of violence against emergency nurses during the previous two years was 73.7%, and the most common type of violence was verbal abuse. The perpetrators in 88.3% of cases were patients' family members or relatives [12].

Moreover, a study in China (2021) indicated that 29.1% of violence occurred against emergency department clinicians, and a negative correlation was reported between WPV and quality of care [13]. In another study in Turkey (2022), the prevalence of violence against emergency nurses was 90%, and verbal abuse was the most common type of violence. The perpetrators of most of the violence were the patients' relatives, and the cause of violence in most cases was the long waiting time to receive care [14]. The results of a study in Taiwan (2021) showed that the prevalence of violence against emergency nurses was 54%, and the most common type of violence was mental violence [1].

WPV may increase the risk of physical, psychological, and behavioral problems in nurses, including sleep disorders, fatigue, pain, fear, anxiety, depression, post-traumatic stress disorder, substance abuse, communication problems, and job burnout [7, 15], also aggravating the nurse shortage problem [1].

Since WPV is a growing phenomenon and a major threat to nurses [16], it is necessary to take practical measures to reduce violence in this walk of life, and the first step in this direction is to know the prevalence of violence [9, 17]. In this regard, several studies that have investigated the prevalence of WPV and its related factors in emergency nurses in Iran [2, 5, 6] and around the world [1, 3, 14, 18] have reported different results. This difference may be related to cultural differences and psychological characteristics of patients and their relatives, as well as the nurses' behavioral and emotional balance [1]. Accordingly, the present study was conducted to investigate the prevalence of WPV and its related factors in emergency nurses.

This study sought to answer the following questions: (1) What is the prevalence of physical and verbal violence against emergency nurses during the past 12 months? (2) What are the factors related to violence against emergency nurses? (3) What is the response of emergency nurses to violence? (4) What are the strategies for preventing and managing WPV? and (5) What are the characteristics of WPV?

## 2. Methods

### 2.1. Study Design

This research is a cross-sectionaldescriptive-analytical study. In this type of study, the outcome and exposure variables are collected simultaneously; therefore, it is not possible to determine the cause-and-effect relationships between these variables [19]. The Strengthening the Reporting of Observational Studies in Epidemiology (STROBE) statement was used to report the results [20].

### 2.2. Sample and Sampling Method

The study population included nurses (*n* = 300) working in the emergency departments of seven hospitals affiliated to Kermanshah University of Medical Sciences (KUMS). Considering the prevalence of violence against nurses in the study of Honarvar et al., with a rate of about 90% [3], 5% accuracy, and a 95% confidence level, the sample size was estimated to be 143 nurses, and with 5% nonresponse, the sample size was increased to 150. The inclusion criteria were a bachelor's degree or higher in nursing and at least one year of experience in the emergency department. For sampling, first, the sampling frame of each hospital was received from the nursing office. The participants were recruited by stratified random sampling method. In this method, the research population is divided into several subgroups or strata based on common characteristics. Then, within each group, the necessary sample size is randomly selected. In the current study, seven emergency departments of KUMS-affiliated hospitals formed the strata. Within each stratum, simple random sampling was performed using a table of random numbers. Incomplete completion of the questionnaire was regarded as the exclusion criterion. It should be pointed out that no one was excluded from the study.

### 2.3. Measurement Instrument

The study tool was a researcher-made questionnaire that was designed using previous studies [2–5]. The questionnaire consisted of two parts. The first part was about the participants' demographic information, including age, gender, marital status, work experience, and type of employment. The second part consisted of 15 questions on the frequency of physical and verbal violence during the past 12 months, the availability of instructions on reporting the violence, the level of concern about the occurrence of violence in the workplace, the perpetrator's characteristics, the place of violence, the time of violence, the perceived causes of violence, and the nurses' response to violence. At the end of the questionnaire, an open-ended question was placed, and the nurses were asked to express their ways of preventing and managing WPV.

Questions were of the multiple choice, yes/no, likert, and open-ended types. Some of the questions were: “Have you been physically abused in the past twelve months? Yes/No,” “What was the gender of the perpetrator? Male/female,” “Who was the perpetrator? Patient, patient's relatives, patient's friends, patient's family, and colleague,” “Does your workplace have guidelines for reporting cases of violence? Yes/No,” “How concerned are you with workplace violence? Never, very little, little, moderately, very much,” and “In your opinion, what are the ways to prevent and manage workplace violence against nurses?” To score the questionnaire, the simple and relative frequency and standard deviation of the responses were calculated.

Quantitative and qualitative content validity methods were used to evaluate the validity of the measurement tool. In the qualitative section, the tool was provided to 15 faculty members, their corrective opinions regarding different parts of the questionnaire were obtained, and necessary corrections were made in the questionnaire. In the qualitative part, the content validity index and content validity ratio were calculated, which were equal to 0.82 and 0.72, respectively. A four-point Likert scale was used to calculate CVI, and experts were asked to choose one of the options of completely relevant, relevant, approximately relevant, and irrelevant for each question. The number of experts who chose approximately relevant and irrelevant options was divided by the total number of experts [21]. A CVI higher than 0.79 was considered appropriate. CVR was calculated using a three-point Likert scale, including the options “essential,” “useful, but not essential,” and “not necessary.” Experts were asked to choose one of the options for each question. The number of experts who chose the answer “necessary” was included in the calculation formula of CVR [21]. A CVR higher than 0.49 was considered satisfactory.

The test-retest method was used to evaluate the reliability. For this purpose, the questionnaire was completed by 30 nurses working in the emergency department with an interval of one week, and a correlation coefficient of 0.78 was obtained. It should be noted that these nurses were not included in the study.

### 2.4. Data Collection Method

To collect data, the researcher referred to the nursing offices of hospitals affiliated to KUMS and received a list of nurses working in the emergency department. Then, according to the number of nurses in each hospital, the required sample size was selected. Next, the selected nurses were visited according to their work schedule. At first, the objectives of the study were stated for nurses, and if they were willing, they were included in the study. Then, a questionnaire was given to the participants and collected after completion. If the participant did not agree to participate in the study, the person before or after him/her in the list of names would be replaced.

### 2.5. Statistical Analysis

Data were analyzed by SPSS V.18 software using descriptive and inferential statistics. In the descriptive part, the frequency, mean, and standard deviation indices were used. In the analytical section, the logistic regression analysis was used to determine the factors related to physical and verbal violence. The desired significance level was less than 0.05. The quantitative content analysis method was used to analyze the samples' answers to the open-ended question. In this method, data analysis is done based on frequency and percentages. In the current study, the frequency of WPV prevention and management strategies was determined.

### 2.6. Ethical Considerations

The Ethics Committee of Kermanshah University of Medical Sciences approved the study with the code KUMS.REC.1395.494. The goals of the study were stated for all participants, and they were assured that their details and responses would remain confidential. Written informed consent was obtained from all participants.

## 3. Results

A total of 150 nurses participated in this cross-sectional study, with a response rate of 100%. All participants were nurses, and 77.3% (*n* = 116) contractual nurses. Most of them were female (*n* = 88, 58.7%) and single (*n* = 80, 53.3%), with an age of less than 30 years (*n* = 106, 70.7%) and a work experience of 1–5 years (*n* = 109, 72.7%) (Table 1).

The frequency rates of physical and verbal violence during the past 12 months were equal to 62% (95% CI: 53.8, 69.5) (*n* = 93) and 94.7% (95% CI: 89.6, 97.3) (*n* = 142), respectively. In both physical (*n* = 40, 43.0%) and verbal violence (*n* = 101, 71.1%), the most common perpetrator was the patient's family. The most common perpetrator gender was male in both physical (*n* = 83, 89.2%) and verbal violence (*n* = 125, 88.0%). From the nurses' point of view, the most common perceived causes of violence were lack of nurses (*n* = 137, 96.0%), long waiting times (*n* = 131, 92.0%), deterioration of patients' clinical conditions (*n* = 120, 84.0%), and weakness in nurses' communication skills (*n* = 105, 73.5%). In both types of physical (*n* = 46, 49.5%) and verbal (*n* = 57, 40.2%) violence, the nursing station was the most common place of violence. Most physical violence (*n* = 53, 57.0%) and verbal violence (*n* = 50, 35.2%) occurred in night shifts (Table 2).

The majority of participants (*n* = 124, 82.7%) were highly concerned about violence and aggression in the workplace. Most nurses (*n* = 124, 82.7%) stated that there are no specific guidelines for reporting WPV. In response to physical violence, 22.6% (*n* = 14) of nurses stated that they reported the event to their superiors, and 16.1% (*n* = 15) did not take any action. Regarding verbal violence, 21.1% (*n* = 30) of nurses stated that they did not take any action, and only 22.6% (*n* = 14) reported it to their superiors. Further, 69% (*n* = 98) of the participants believed that reporting violence was useless (Table 1).

Based on logistic regression analysis, no statistically significant relationship was found between physical and verbal violence and participants' demographic variables, including gender, age, marital status, type of employment, and work experience (only in the case of physical violence, which entered the model) (Tables 3 and 4).

In response to an open-ended question, ten nurses mentioned two to three ways to prevent and manage WPV. All the answers were explained in four items, including adequate manpower supply (*n* = 8, 80%), holding periodic communication skills workshops (*n* = 6, 60%), prosecution of cases of violence (*n* = 5, 50%), and review of antiviolence policies (*n* = 3, 30%).

## 4. Discussion

WPV against nurses is a global concern [3]. This study was conducted to investigate the prevalence of WPV and its related factors in emergency department nurses. In the current study, 94.7% and 62.0% of nurses reported experiencing verbal and physical violence in the previous year, respectively. Evidence shows that verbal violence among emergency department nurses over a period of one year is between 66.4% and 100%, and the frequency of physical violence varies from 16.7% to 74.9% [4].

A study showed that about 90.0% of emergency department nurses in 13 general hospitals in Beijing, China, had experienced WPV in the previous year [22]. In a study in Shiraz, Iran (2019), 89.6% of nurses had experienced at least one type of violence in the past year, and the verbal harassment rate was about three times greater than physical violence [3]. In another study in Italy (2020), 96.3% of nurses stated that they had suffered from WPV in the past 12 months [18]. Evidence suggests that nurses working in emergency departments are at the risk of violence, which can affect their physical and mental health as well as the quality of nursing care provided.

In line with previous studies [18, 23–27], the perpetrator in both physical and verbal violence was the patient's family. However, in a study conducted among Indonesian nurses, the perpetrators in 43.5% of physical violence and 55.6% of verbal violence were patients and their relatives, respectively [3, 28]. Violence by the patient's family can be related to their concerns about the patient's condition, unfulfilled expectations, and inappropriate psychological conditions.

In the present study, the most common sex of the perpetrator was male in both physical and verbal violence, which is consistent with the results of previous studies [3, 18, 28]. There are several factors involved in this gender difference, including the cultural characteristics of the society and the physical abilities of men.

Contrary to previous studies [2, 29, 30], in the present study, most of the nurses who were subjected to physical and verbal violence were female. But in the study of Saleh et al. (2020) in Mashhad, Iran, most of the abused nurses were male [5]. Nurses, whether male or female, are not immune to violence. Contradictions in the results of studies can be related to differences in sample size and organizational research environments.

In line with previous studies [23, 28, 31, 32], most of the physical and verbal violence occurred during the night shift, which may be related to the increased number of patients referred to the emergency department due to the closure of specialized clinics and the absence of a management team.

In the present study, the nursing station was the most common site of physical and verbal violence. In a study in Iran (2010), the most common sites of verbal and physical violence were the nursing station (49.1%) and the patient's bedside (48.5%), respectively [2]. However, in Italy (2020), the most common site of violence was around the triage area (38.5%) [18], but in Taiwan (2020) and Oman (2020), most cases of violence occurred in the treatment area (61.2% and 63.2%, respectively) [23, 33]. Although there is a possibility of violence in any part of the emergency department, the probability of violence in this area is higher due to the importance of the nursing station in patient management.

In line with previous studies [2, 25], most nurses reported the violence to their superiors, but most believed that reporting the violence was useless. However, in a study in Indonesia (2018), 92.1% of nurses were reluctant to report violence, found it useless, and feared the negative consequences of reporting [28]. Unfortunately, evidence suggests that hospital managers are not serious about pursuing and investigating cases of violence [3]. Lack of trust in the reporting team and fear of retaliation are other reasons for not reporting violence [2]. Reporting violence has positive effects on nurses as well as hospitals and can be helpful in adopting better policies to prevent future violence.

In the present study, the most common causes of physical and verbal violence were a lack of nurses, long waits, patients' inadequate clinical conditions, and nurses' poor communication skills. In other studies, the causes of violence against nurses have been reported to include inappropriate conditions of the patient and their family, unrealistic expectations of patients, noncompliance with hospital rules by patients and their companions, poor communication skills of nurses and patients, a lack of nurses, a lack of facilities, burnout of nurses, and a lack of security forces [2, 3, 28]. Clearly, eliminating the underlying causes of violence plays an important role in preventing WPV, and it seems that manpower supply has a more effective role.

The results showed no statistically significant relationship between WPV and nurses' age. However, in a study conducted on Iranian nurses (2020), the age group of 20–30 years was more exposed to verbal and physical violence than other age groups [5]. The results of a study (2019) in Italy showed that the rate of violence against nurses decreased as the age increased, so that the age group of 40–60 years experienced the least amount of physical and verbal violence compared to the age group under 40 years [4]. With an increase in age, nurses are expected to have more communication skills and therefore play a more effective role in managing and preventing violent incidents.

Consistent with the results of Pandey et al. in Nepal [34], in the current study, no statistically significant relationship was found between the occurrence of violence and nurses' type of employment. However, in a number of studies, a statistically significant relationship has been reported between the occurrence of violence and the type of employment. In this regard, in the study of Honarvar et al. in Shiraz, Iran, more verbal violence occurred among contract nurses [3]. In a study by Jafree in Pakistan, contractual employment was one of the risk factors for experiencing violence among nurses [35]. However, the authors of the present study believe that violence can affect all nurses, regardless of their type of employment.

The results showed no statistically significant relationship between violence and nurses' marital status, which is in line with the results of the study of Pandey et al. in Nepal [34]. But in the study of Saleh et al. In Iran, the rate of physical and verbal violence was higher among married nurses [5].

Similar to previous studies [5, 23, 36], the results of the present study showed no statistically significant relationship between nurses' work experience and violence. In a study in Egypt (2017), 68% of abused nurses had less than 50 years of work experience [37]. But in the study of Choi and Lee, the frequency of violence was higher in nurses with more than 5 years of work experience than in others [38]. The results of a study in Italy (2019) also showed that nurses with a history of less than 10 years had the highest rate of verbal and physical violence [4]. Regardless of the differences in the results of the studies, nurses with more work experience are expected to have better communication skills, to act as role models for less experienced nurses, and to prevent the occurrence of violence.

### 4.1. Study Limitations

In this study, data were collected by the self-report method, which might have affected the results. The second limitation is the possibility of recall bias, which might also have affected the accuracy of the results. In this type of bias, the participants may not remember all the violent events and their details. In this study, the occurrence of violence was based solely on the participants' responses to a yes/no question. Also, different types of verbal/physical violence were not investigated.

## 5. Conclusion

Our results emphasize the high prevalence of violence against emergency department nurses. Most cases of violence were committed by patients during night shifts at the nursing station. Although most nurses reported the violence to their supervisor, they found it useless. Considering the important role of nurses in the health care system, their safety in the workplace must be guaranteed. It is necessary to adopt a global approach to reduce violence in the nurses' workplace. In this regard, health policymakers should take the necessary measures to eliminate violence against nurses and provide a safe work environment with the cooperation of experts and the mass media. Adopting security measures in emergency departments can also play an important role in reducing violence. In this regard, installing closed-circuit cameras in nursing stations and waiting rooms, using fixed security forces, and physically inspecting people for weapons are necessary measures. Also, holding training workshops on the prevention and management of workplace violence can be useful for emergency nurses. Similar studies in other care sectors as well as longitudinal studies are required to investigate the effect of intervention measures on the frequency of workplace violence.

## Figures and Tables

**Table 1:** Demographic characteristics of the nurses according to the type of violence.

Variables		Total	Physical violence		Verbal violence	
			*n* (%)		*n* (%)	
			Yes	No	Yes	No
Sex	Male	62 (41.3)	37 (39.8)	25 (43.9)	59 (41.5)	3 (37.5)
	Female	88 (58.7)	56 (60.2)	32 (56.1)	83 (58.5)	5 (62.5)

Age (year)	≤30	106 (70.7)	63 (67.7)	43 (75.4)	99 (69.7)	7 (87.5)
	>30	44 (27.3)	30 (32.3)	14 (24.6)	43 (30.3)	1 (12.5)

Marital status	Single	80 (53.3)	53 (57.0)	27 (47.4)	78 (54.9)	2 (25.0)
	Married	70 (46.7)	40 (43.0)	30 (52.6)	64 (45.1)	6 (75.0)

Employment type	Formal	34 (22.7)	21 (22.6)	13 (22.8)	33 (23.2)	1 (12.5)
	Contractual	116 (77.3)	72 (77.4)	44 (77.2)	109 (76.8)	7 (87.5)

Job experience (Year)	1–5	109 (72.7)	69 (74.2)	40 (70.2)	106 (74.6)	3 (37.5)
	6–10	31 (20.7)	17 (18.3)	14 (24.6)	26 (18.3)	5 (62.5)
	≥11	10 (6.7)	7 (7.5)	3 (5.3)	10 (7.0)	0 (0.0)

Concerns about workplace violence	Low	9 (6.0)	6 (6.5)	3 (5.3)	9 (6.3)	0 (0.0)
	Medium	17 (11.3)	9 (9.7)	8 (14.0)	15 (10.6)	2 (25.0)
	High	124 (82.7)	78 (83.9)	46 (80.7)	118 (83.1)	6 (75.0)

Existence of violence reporting guidelines	Yes	26 (17.3)	17 (18.3)	9 (15.8)	24 (16.9)	2 (25.0)
	No	124 (82.7)	76 (81.7)	48 (84.2)	118 (83.1)	6 (75.0)

“Age, gender, work experience, marital status, job experience, concerns about workplace violence, and type of employment.”

**Table 2:** Characteristics of violent incidents in emergency department nurses.

Variables		Physical violence	Verbal violence
		*n* (%)	*n* (%)
Exposure to violence over the past year	Yes	93 (62.0)	142 (94.7)
	No	57 (38.0)	8 (5.3)

Source of violence	Patient	4 (4.3)	17 (12.0)
	Patient's family	40 (43.0)	101 (71.1)
	Staff member	22 (23.7)	6 (4.2)
	Supervisor/head nurse/manager	14 (15.1)	17 (7.2)
	Other	13 (13.9)	2 (5.5)

Perpetrator gender	Male	83 (89.2)	125 (88.0)
	Female	10 (10.8)	17 (12.0)

Place of violence	Patient room	19 (20.4)	48 (33.8)
	Waiting room	4 (4.3)	4 (2.8)
	Treatment room	4 (4.3)	33 (23.2)
	Nursing station	46 (49.5)	57 (40.2)
	Other	20 (21.5)	—

Violence in the work shift	Morning	17 (18.3)	45 (31.7)
	Evening	23 (24.7)	47 (33.1)
	Night	53 (57.0)	50 (35.2)

**Table 3:** Relationship between demographic variables and physical violence.

Variables		Physical violence					
		Crude OR^a^	95% CI^b^	*P* value	aOR^c^	95% CI	*P* value
Sex	Female	Ref	—		Ref		
	Male	1.95	0.97, 3.88	0.059	1.77	0.86, 3.64	0.123

Age (years)	≤30	Ref			Ref		
	>30	1.46	0.69, 3.08	0.316	2.86	0.62, 13.21	0.178

Marital status	Married	Ref			Ref	—	
	Single	1.17	0.61, 2.27	0.637	1.13	0.54, 2.38	0.748

Employment type	Official	Ref			Ref		
	Contractual	1.01	0.46, 2.22	0.974	2.15	0.43, 10.67	0.347

Job experience (years)	≥11	Ref			Ref		
	6–10	0.68	0.17, 2.8	0.596	1.02	0.13, 8.04	0.986
	1–5	0.68	0.15, 3.1	0.620	0.82	0.17, 3.99	0.803

*Note.* ^a^Odds ratio; ^b^confidence interval; ^c^adjusted odds ratio.

**Table 4:** Relationship between demographic variables and verbal violence.

Variables		Verbal violence					
		Crude OR^a^	95% CI^b^	*P* value	aOR^c^	95% CI	*P* value
Sex	Male	Ref			Ref		
	Female	0.84	0.19, 3.67	0.821	1.03	0.23, 4.67	0.972

Age (years)	≤30	Ref			Ref		
	>30	3.04	0.36, 25.47	0.305	3.33	0.15, 72.69	0.445

Marital status	Married	Ref			Ref		
	Single	3.65	0.71, 18.74	0.120	3.49	0.66, 18.49	0.142

Employment type	Official	Ref			Ref		
	Contractual	0.47	0.06, 3.97	0.490	1.29	0.06, 28.96	0.874

*Note.* ^a^Odds ratio; ^b^confidence interval; ^c^adjusted odds ratio.

## Data Availability

The identified datasets analyzed during the current study are available from the corresponding author on a reasonable request.
